# A Simplified and Robust Activation Procedure of Glass Surfaces for Printing Proteins and Subcellular Micropatterning Experiments

**DOI:** 10.3390/bios12030140

**Published:** 2022-02-25

**Authors:** Tina Karimian, Roland Hager, Andreas Karner, Julian Weghuber, Peter Lanzerstorfer

**Affiliations:** 1 School of Engineering, University of Applied Sciences Upper Austria, 4600 Wels, Austria; tina.karimian@fh-wels.at (T.K.); roland.hager@fh-wels.at (R.H.); julian.weghuber@fh-wels.at (J.W.); 2 School of Engineering, University of Applied Sciences Upper Austria, 4020 Linz, Austria; andreas.karner@fh-linz.at; 3 FFoQSI GmbH, Austrian Competence Center for Feed and Food Quality, Safety & Innovation, 3430 Tulln, Austria

**Keywords:** micropatterning, micro-contact printing, fluorescence microscopy, live cell analysis

## Abstract

Depositing biomolecule micropatterns on solid substrates via microcontact printing (µCP) usually requires complex chemical substrate modifications to initially create reactive surface groups. Here, we present a simplified activation procedure for untreated solid substrates based on a commercial polymer metal ion coating (AnteoBind^TM^ Biosensor reagent) that allows for direct µCP and the strong attachment of proteins via avidity binding. In proof-of-concept experiments, we identified the optimum working concentrations of the surface coating, characterized the specificity of protein binding and demonstrated the suitability of this approach by subcellular micropatterning experiments in living cells. Altogether, this method represents a significant enhancement and simplification of existing µCP procedures and further increases the accessibility of protein micropatterning for cell biological research questions.

## 1. Introduction

Depositing biomolecules onto solid substrates in regular 2D patterns with micrometer resolution, also known as molecular printing or, more commonly, as protein micropatterning, has found widespread use in academic laboratories, and multiple applications for biomedical and cell biological research have emerged [1,2,3,4,5,6,7,8]. In this regard, many different methodologies have been developed in the recent years, whereas the fabrication of biomolecule micropatterned (and even nanopatterned) solid substrates is mainly depending on the intended application and available lab infrastructure. Basically, those techniques can be classified into direct and indirect deposition strategies.

The indirect strategies include methodologies such as the widely used photolithography [9], laser microablation [10], colloidal lithography [11], di-block copolymer micelle nanolithography [12] or di-block copolymer self-assembly [13]. Direct protein deposition can be realized by approaches such as dip-pen lithography (DPL) [14], microchannel cantilever spotting (µCS) [15], polymer pen lithography (PPL) [16], microfluidic patterning [17], fluidic force microscopy (FluidFM) [18] and soft lithography via microcontact printing (µCP) [19]. Within those tools, µCP (especially in combination with poly(dimethylsiloxane) (PDMS) stamps) is the most convenient and widely used method for protein micropatterning. Protein patterning by µCP provides unique features compared with all other sophisticated patterning technologies as it is: (i) highly reproducible and robust, (ii) easy to perform, (iii) extremely fast, (iv) modularly expandable (with respect to feature size and printed protein), (v) comparatively cheap, and (vi) independent of special lab equipment. Variations, core advantages and methodological limitations of µCP have been reported and discussed in various recent studies [17,19,20,21,22,23]. 

Though comparatively simple in implementation and execution, µCP still requires complex chemical modifications of the substrate to increase surface energy and to generate functional groups (e.g., carboxyl, amine, hydroxyl or epoxy groups) for the direct covalent attachment of biomolecules. Functional chemical moieties are typically introduced using methodologies such as plasma treatment, UV irradiation and monolayer self-assembly [24,25,26]. Alternatively, customized substrates carrying a wide range of functional coating chemistries are available from various companies. However, a direct company supply is often associated with long waiting periods, high purchase quantities and variations in surface quality, resulting in rather expensive substrates for subsequent µCP and intended applications.

Most recently, a nanocontact printing approach for the generation of protein nanopatterns (80 nm feature size) on polymer–metal ion-coated substrates was reported using a Mix&Go surface chemistry (AnteoBind^TM^ Biosensor reagent) and a two-layer stamp architecture (X-PDMS) [27]. The applied polymer–metal ion coating does not react with water and allows for the strong attachment of proteins via avidity binding. In this regard, such coated surfaces have been reported for various different applications, ranging from electrochemical immunosensors [28,29,30], antibody immunoassay [31], amperometric immunoassay [32] and extracellular vesicle detection [33]. Furthermore, metal-polyphenol coatings and micropatterns have been recently reported for simple biomolecule binding [34] and platelet adhesion [35].

Here, we present an extension of the µCP method for the simplified and robust fabrication of 2D protein micropatterns. We exemplify our approach by creating microstructured streptavidin- and antibody-patterned surfaces with feature sizes of 3 µm. We provide a detailed protocol for the implementation of the Mix&Go surface chemistry for glass substrates, identify optimum working concentrations and demonstrate the biocompatibility by subcellular micropatterning experiments in living cells.

## 2. Materials and Methods

### 2.1. Reagents and DNA Constructs

Bovine serum albumin, streptavidin, polydimethylsiloxane (PDMS, SYLGARD^®^ 184), AnteoBind^TM^ Biosensor reagent, FITC-conjugated avidin, and Tween-20 were purchased from Sigma-Aldrich (Schnelldorf, Germany). 2-(N-morpholino)ethanesulfonic acid (MES) was purchased from Carl Roth (Karlsruhe, Germany). Cy5-conjugated streptavidin and Zenon IgG Labeling Kit was obtained from ThermoFisher Scientific (Waltham, MA, USA). BSA-Cy5 was purchased from Protein Mods (Madison, WI, USA). Biotinylated antibodies were obtained from Antibodies Online (Herford, Germany). Ninety-six-well plastic castings were obtained from Greiner Bio-One GmbH (Frickenhausen, Germany). NEXTERION glass coverslips (110 mm × 74 mm, 175 ± 20 µm thickness) were from Schott GmbH (Jena, Germany). Biotin-labeled human fibrinogen was purchased from Innovative Research (Novi, MI, USA).

### 2.2. Glass Substrate Activation

Glass coverslips were initially cleaned with EtOH and dH_2_O, dried under a stream of air (or nitrogen) and incubated with 15 mL AnteoBind^TM^ Biosensor reagent (with indicated concentrations) for 30 min. Subsequently, excess reagent was carefully removed from the surface and the coverslip was again washed with dH_2_O and dried. Coated glass substrates were immediately used for µCP.

### 2.3. Preparation of Large-Area PDMS Stamps

A PDMS prepolymer was mixed in a ratio of 10:1 (*w*/*w*, precursor:curing agent) and degassed in a desiccator for 30 min to remove air bubbles. The PDMS mixture was then poured on a silanized wafer (Delta Mask, Enschede, The Netherlands) containing an array of round-shaped pillars with a feature size and depth of three micrometers. The mixture on the wafer was degassed again, cured for 2 h at 80 °C and, finally, peeled off from the wafer for µCP.

### 2.4. Preparation of Protein Micropatterns by µCP

Prior to µCP, the PDMS stamp was washed with EtOH and dH_2_O, followed by drying under a stream of air (or nitrogen). The stamp was then incubated for 1 h at room temperature with a BSA or BSA-Cy5 solution (with indicated concentrations) in the dark. Subsequently, the stamp was washed with PBS and dH_2_O, dried again, and placed by its own weight upside down on the activated glass substrate. The stamp was peeled off after 30 min of incubation and the BSA-patterned substrate was bonded to a 96-well plastic casting using an adhesive tape (3M, laser cut to 96-well layout). The resulting reaction chambers were subsequently incubated with 70 µL streptavidin or streptavidin-Cy5 solution (50 µg/mL in 25 mM MES, pH 6) for 20 min at room temperature. Protein-patterned surfaces were washed three times with PBST (pH 7.4 with 0.05% Tween-20 (*v*/*v*)) and imaged by fluorescence microscopy.

### 2.5. Cell Culture and Transfection

HeLa cells were obtained from ATCC, cultured in RPMI medium supplemented with 10% FBS and 1% penicillin/streptomycin (all PAN-Biotech GmbH, Aidenbach, Germany) and grown at 37 °C in a humidified incubator with 5% CO_2_. Cells were transiently transfected with fluorescent-fusion constructs using jetOPTIMUS DNA transfection reagent (Polyplus transfection, Illkirch, France), according to the manufacturer’s protocol. A total of 24–48 h after transfection, cells were used for subcellular micropatterning experiments.

### 2.6. Subcellular Micropatterning Experiments in Living Cells

For live cell micropatterning experiments, streptavidin-patterned surfaces were further modified by incubating biotinylated bait antibodies (10 µg/mL, as indicated) for another 20 min at room temperature. Antibody-functionalized chambers were finally washed with PBS (three times) and bait-protein expressing cells were grown on the patterned surfaces for at least 3–4 h prior to fluorescence imaging.

### 2.7. Fluorescence Microscopy

TIRF microscopy was carried out on a microscopy set-up as used in a previous study [36].

### 2.8. Atomic Force Microscopy

AFM imaging was performed at room temperature on a JPK NanoWizard 4 (Bruker, MA, USA) operated in tapping mode and using MSNL cantilevers (Bruker, MA, USA). Open source Gwyddion software (version 2.60, http://gwyddion.net/) was used for image processing [37].

### 2.9. Image Analysis and Statistical Evaluation

Initial imaging recording was supported by the Olympus XcellenceRT software package (version 1.1). Images were exported as TIFF frames and fluorescence contrast analysis was performed using the Spotty framework (version 3.7, https://bioinformatics.fh-hagenberg.at/bin_typo3/htdocs/fileadmin/user_upload/Downloads/spotty.html) as described previously [36,38].

For significance testing, an unpaired t-test was used to compare two experimental groups, whereas comparison of more than two different groups was performed using one-way ANOVA, which was followed by Tukey’s multiple comparisons test. All data transformation and statistical comparisons were carried out in GraphPad Prism software (version 9).

## 3. Results and Discussion

### 3.1. Simplified Procedure for the Fabrication of Protein Micropatterned Glass Substrates

**Figure 1:**
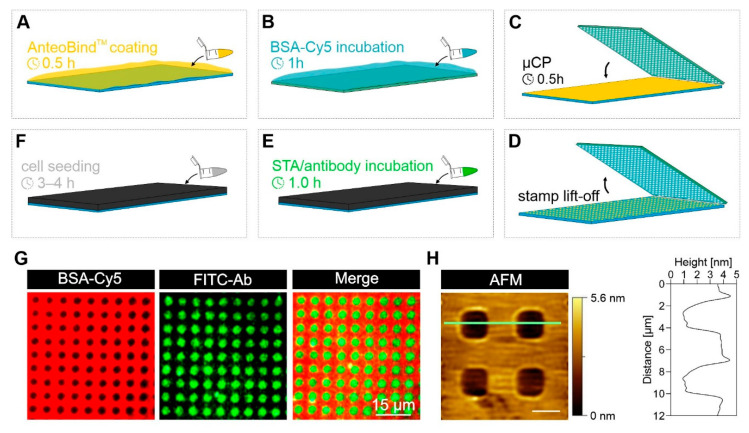
Schematic workflow of simplified large-area protein micropatterning on activated glass substrates. In short, untreated glass substrates are activated by coating with AnteoBind^TM^ reagent (**A**). In parallel, a large-area PDMS stamp is incubated with BSA (or BSA-Cy5) solution for surface passivation (**B**). The stamp is subsequently placed onto the substrate, resulting in a transfer of the micron-scale BSA grid for surface passivation (**C**,**D**). After the stripping of the stamp, the patterned glass substrate is bonded with a 96-well plastic casting. Next, streptavidin and biotinylated antibodies are sequentially incubated (**E**). In a final step, cells are seeded onto the antibody-patterned surfaces for fluorescence microscopy analysis (**F**). Exemplary TIRF images of BSA-Cy5 printed surfaces with FITC-labeled antibody patterns (**G**). AFM image of micron-scale BSA grid and respective line profile. Scale bar = 3 µm (**H**).

The principle of our surface preparation is shown in Figure 1A–F, exemplified by large-area surface patterning for increased experimental throughput. A simplified scheme of the µCP process is depicted in Figure S1. Following this workflow, the micron-scale protein-patterned glass substrate was generated by printing a biocompatible and chemically stable background protein possessing good surface passivation properties onto the AnteoBind^TM^ precoated glass. As shown in our previous work [36,39], BSA meets those demands and was used throughout this study. AFM images of the BSA grid revealed an almost defect-free surface passivation (Figure 1H), with an average height of the printed BSA of ~3–4 nm, which corresponds to a BSA monolayer formed upon µCP. After µCP, the pre-patterned glass substrate was manually bonded to a multi-well plastic casting resulting in a modular ready-to-use micropatterning platform. The unblocked 3 µm patterns were subsequently filled with streptavidin, followed by the incubation of biotinylated antibodies (Figure 1G, TIRF microscopy images of BSA-Cy5 grid and FITC-labeled antibodies are shown for illustration) and the seeding of cells expressing fluorescence fusion proteins of interest. The antibody will bind to the extracellular domain of a membrane-anchored protein (e.g., receptor; also termed as bait protein), resulting in a rearrangement of the bait in the cell membrane into an ordered array according to the micron-scale antibody pattern. We and others have used similar assays (although with methodological variations) to investigate cell signaling and cell membrane receptor-dependent protein–protein interactions (PPIs) [40,41,42,43,44,45,46,47]. Most recently, we further developed the approach enabling the subcellular dynamic immunopatterning of cytosolic protein complexes [36]. The use of this platform is not only restricted to antibodies, rather, it enables fast and easy modular surface functionalization using different bait-capturing biomolecules such as specific ligands [46], ligand-decorated multi-scale origami structures [39] and multivalent chelator nanotools [47].

By adapting our micropatterning assay to the described polymer–metal ion coating, we attempted to create very stable and active surfaces, which are capable of binding many classes of biomolecules and serve as biointerfaces for subcellular micropatterning experiments in living cells.

### 3.2. Characterization of Protein Binding on Micropatterned Substrates

**Figure 2:**
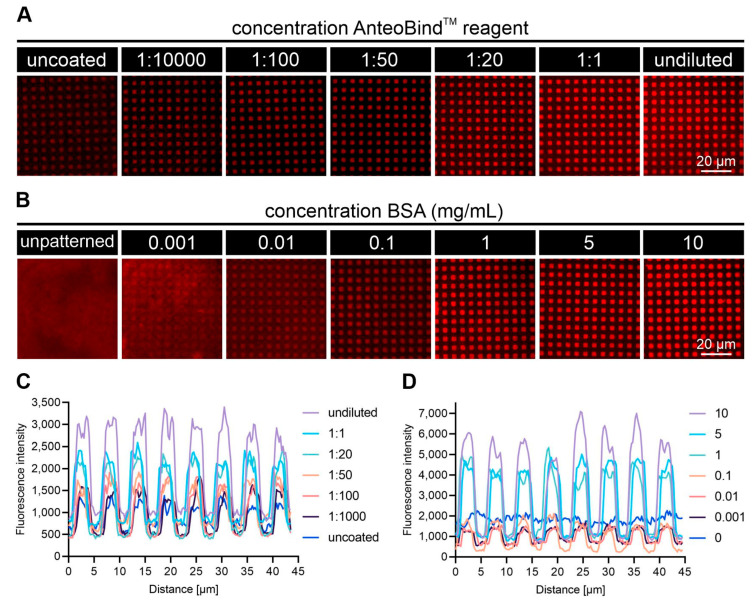
Characterization of AnteoBind^TM^ coating for protein micropatterning via µCP. (**A**) Glass substrates were precoated with indicated concentrations of AnteoBind^TM^ reagent (diluted in ddH_2_O) prior µCP with BSA-inked (5 mg/mL) PDMS stamps. BSA-patterned substrates were further modified with Cy5-labeled streptavidin (50 µg/mL). Binding of STA-Cy5 to uncoated glass surfaces was used as control. (**B**) Glass substrates were precoated with AnteoBind^TM^ reagent (1:20 in ddH_2_O) prior µCP with BSA-inked (indicated BSA concentrations) PDMS stamps. BSA-patterned substrates were further modified with Cy5-labeled strep(tavidin (50 µg/mL). Binding of STA-Cy5 to coated but unpatterned surfaces was used as control. (**C**) Representative line profiles of STA-Cy5 fluorescence signal at indicated AnteoBind^TM^ dilution as shown in A. (**D**) Representative line profiles of STA-Cy5 fluorescence signal at indicated BSA concentration as shown in B.

To elaborate on the applicability of the polymer–metal ion coating for protein micropatterning via µCP, we functionalized untreated glass coverslips using the procedure as described in Figure 1. In a first step, we identified optimum working concentrations of the AnteoBind^TM^ coating reagent (Figure 2A,C). For this purpose, glass substrates were covered by different concentrations of the polymer–metal ion reagent followed by µCP of the micron-scale BSA grid (5 mg/mL) and subsequent incubation of STA-Cy5, whereas the quality of BSA transfer was evaluated by means of STA-Cy5 fluorescence signal. Figure 2A shows representative TIRF microscopy images of STA-Cy5-patterned glass surfaces that were precoated with indicated AnteoBind^TM^ reagent concentrations. Representative line profiles of the respective STA-Cy5 signals are shown in Figure 2C. Non-specific binding of STA-Cy5 in BSA-patterned grid areas was comparably reduced to a minimum for surfaces coated with pure reagent down to a dilution step of 1:20. However, the highest levels of specific STA-Cy5 enrichment inside the non-passivated patterns were detected on substrates with pure AnteoBind^TM^ reagent coating, followed by slightly reduced fluorescence signals down to a concentration of a 1:20 dilution. AnteoBind^TM^ reagent concentrations below 1:20 delivered significantly lower levels of STA-Cy5 patterning, whereas the highest background signal in combination with the lowest STA-Cy5 signal was obtained for glass surfaces without polymer-metal ion coating. Indeed, biomolecule printing and binding without loss of functionality onto untreated glass coverslips has been reported in various studies [44,48,49]. However, exact mechanisms of protein binding on unmodified glass have not been fully understood so far and are mainly dependent on the surface properties themselves, as well as on the adsorption protein [50]. Most importantly, attaching proteins to rather inactive surfaces, such as plane glass, might lead to lower surface passivation, increased unspecific binding, decreased specific binding of the protein of interest, eventual loss of protein function or reduced binding capabilities, and unstable biointerfaces with shortened storage life. Furthermore, it is not entirely clear what holds the polymer–metal ion complex on the bare glass surface. However, it was previously shown that such metal complexes can bind to plain non-irradiated polystyrene surfaces, as well as to untreated cyclic olefin copolymer substrates [31,51]. What is noteworthy, is that untreated glass surfaces were also reported to possess anionic surface properties [52], therefore being able to bind cationic biomolecules, as it might also be the case for the positively charged polymer–metal ion complex. Hence, we speculate that the ‘adsorption forces’ are a mixture of ionic interactions and hydrogen bonds due to surface charges and impurities (e.g., presence of other metal ions, surface oxygen groups, etc.). Eventually, simple diffusion into the porous glass structure might also be a possible explanation.

Knowing the optimum surface coating dilution, we next investigated the impact of the surface passivation by printing a micron-scale BSA grid with varying concentrations (Figure 2B,D). In order to reduce specific costs for surface functionalization (which plays a major role, especially for the large substrates presented here), we picked the 1:20 dilution for surface coating. Figure 2B shows TIRF microscopy images of STA-Cy5-patterned glass surfaces that were precoated with 1:20 AnteoBind^TM^ reagent concentrations and subsequently patterned with a BSA grid using concentrations between 0.001 and 10 mg/mL BSA. Representative line profiles of the respective STA-Cy5 signals are shown in Figure 2D. As a starting point, we chose 1 mg/mL BSA, which we and others have used for surface passivation on epoxysilane-coated glass slides and COP foils in previous studies [36,53]. Nevertheless, higher concentrations of BSA for improved surface passivation coatings have been recently reported [54,55]. Therefore, we increased the BSA amount for µCP up to 10 mg/mL, which indeed resulted in the highest STA-Cy5 signal inside the active patterns (Figure 2D). A slightly lower pattern intensity was observed for 1 and 5 mg/mL BSA-printed surfaces, whereas, already, a tenfold lower concentration led to a substantial reduction in STA-Cy5 pattern enrichment. Almost no specific STA-Cy5 patterning could be detected for 0.001 mg/mL BSA passivated surfaces. Unpatterned, but AnteoBind^TM^ reagent and BSA-coated surfaces were used as a control.

To further characterize the versatility of the implemented coating, we aimed in the printing of different proteins in addition to BSA. As shown in Figure S2, we were able to deposit various proteins (streptavidin, avidin, anti-EGFR IgG antibody, fibrinogen) onto the coated glass substrates by µCP.

The general applicability of the AnteoBind^TM^ reagent for immobilizing biomolecules, and more specifically antibodies, on various surfaces has been reported in several studies [27,28,31,33]. We could unequivocally show that this coating is also a superior and simple strategy for the surface activation, prior to µCP of proteins. Based on the intended application and pursued sensitivity of the assay, the reagent can be used in a wide concentration range, also in combination with common surface passivation steps.

### 3.3. Applicability for Subcellular Micropatterning Experiments

**Figure 3:**
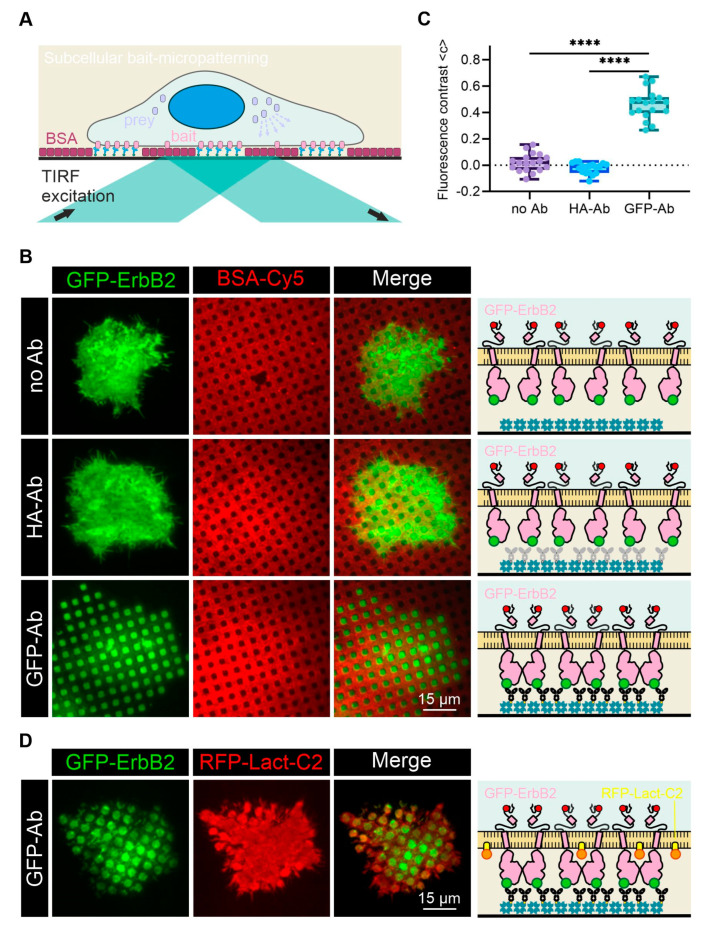
Applicability for subcellular micropatterning experiments. (**A**) Schematic presentation of the subcellular micropatterning assay. Cells are transiently cotransfected with fluorescently labeled bait (and prey) molecules and grown on antibody-patterned surfaces. Upon specific antibody–bait interactions, bait proteins are rearranged in the plasma membrane according to the surface pattern. (**B**) Representative TIRF microscopy images of GFP-ErbB2-expressing cells grown on BSA-Cy5-patterned surfaces consisting of: no antibodies (top), unspecific anti-HA antibodies (middle) and specific anti-GFP antibodies (bottom). (**C**) Box plots show quantitation of GFP contrast of cells grown under conditions as in (**B**) (*n* = 18 cells; **** *p* < 0.0001 for comparison with GFP-Ab). (**D**) Representative TIRF microscopy images of cells coexpressing GFP-ErbB2 and RFP-Lact-C2 grown on anti-GFP antibody-patterned surfaces.

Finally, we demonstrated the applicability of AnteoBind^TM^ reagent-coated and BSA-structured glass surfaces for subcellular micropatterning experiments in living cells. For this purpose, surfaces were further functionalized with streptavidin and anti-GFP antibodies and cells expressing a GFP-fused bait protein were grown on the antibody-patterned substrate. Upon specific antibody–antigen interaction, bait proteins will be rearranged in the plasma membrane according to the micron-scale antibody pattern (Figure 3A). To reduce fluorescence background signal, and to specifically visualize GFP-fused proteins within or near the cell membrane, the fluorescence readout was conducted in total internal reflection mode (TIRF microscopy). As a proof-of-concept bait, we overexpressed GFP-labeled ErbB2 (Erb-B2 receptor tyrosine kinase 2; GFP was fused to the extracellular domain of the receptor) in Hela cells. First, we elaborated on the specificity of bait patterning due to the antibody–antigen interaction (Figure 3B). For the quantitation of the lateral bait distribution, the respective fluorescence signal intensities within and outside the antibody-patterned areas were compared (mean fluorescence contrast <c>) [41]. No rearrangement of GFP-ErbB2 was detected in cells grown on surfaces only functionalized with STA (Figure 3B,C, row 1; <c> = 0.02 ± 0.06) and ‘inert’ anti-HA antibodies (Figure 3B,C, row 2; <c> = −0.02 ± 0.04), respectively. On the contrary, a significant lateral redistribution of GFP-ErbB2 was detected in cells grown on anti-GFP antibody-patterned substrates (Figure 3B,C, row 3), resulting in a mean fluorescence contrast of <c> = 0.46 ± 0.11. This result again confirms robust surface protein patterning. Furthermore, the transfer of the micron-scale antibody pattern into the plasma membrane was only visible in cells facing functional and specific anti-bait antibodies.

As quantitative TIRF microscopy requires a flat interface between the plasma membrane and the patterned substrate to avoid false-positive signals and misinterpretation, we next checked the cell contact surface by coexpressing GFP-ErbB2 and RFP-Lact-C2 (RFP fused with C2 domain of bovine lactadherin) (Figure 3D). The inner-leaflet peripheral protein RFP-Lact-C2 turned out to be a good negative control, as it showed a homogenous membrane distribution in the central regions of GFP-ErbB2-patterned cells. Most importantly, the lack of RFP-Lact-C2 copatterning indicates that bait micropatterning has no measurable influence on plasma membrane curvature.

**Figure 4:**
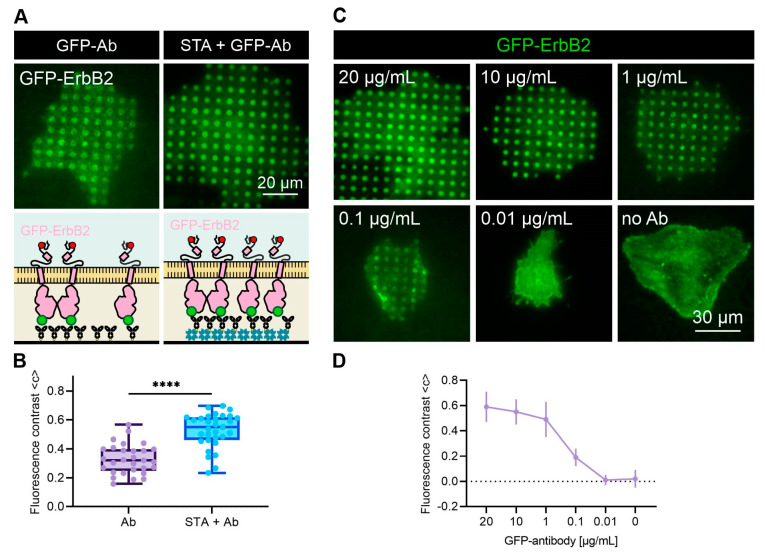
Evaluation of antibody specificity. (**A**) Representative TIRF microscopy images of GFP-ErbB2 expressing cells grown on solely anti-GFP antibody-patterned surfaces (left) and with an additional STA layer in between (right). (**B**) Box plots show quantitation of GFP contrast of cells grown under indicated conditions (*n* > 29 cells; **** *p* < 0.0001 for comparison of the two different conditions). (**C**) Representative TIRF microscopy images of GFP-ErbB2-expressing cells grown on surfaces bearing different concentrations of anti-GFP antibody. (**D**) Dot plot shows quantitation of antibody concentration-dependent mean GFP contrast (*n* = 20 cells per concentration).

AnteoBind^TM^ reagent-coated surfaces have been reported to possess direct antibody binding capabilities with enhanced orientation and functionality, as the polymeric metal ions chelate to available electron-donating groups on synthetic surfaces and biomolecules [31]. We therefore investigated the bait-capturing capability of patterned surfaces that were directly functionalized with anti-GFP antibodies in comparison to surfaces comprising an additional STA layer prior to antibody addition (our “classical” way of antibody patterning) (Figure 4A,B). Indeed, we already found a remarkable GFP-ErbB2 enrichment in cells grown on solely antibody-functionalized substrates (<c> = 0.33 ± 0.10). Interestingly, antibody-induced GFP-ErbB2 patterning could be further enhanced by STA preincubation, resulting in a significantly increased fluorescence contrast value of <c> = 0.53 ± 0.12. These results prove, again, the superior biomolecule-binding properties of this surface coating. Nevertheless, at least for the presented application, an additional streptavidin layer seems to be favorable for enhanced bait-capturing. Reasons for that might be diverse; however, it is fair to speculate that the covalent streptavidin-biotin interaction leads to a more optimized antibody orientation, also preserving its native character. Furthermore, an additional incubation of streptavidin might lead to a better accessibility of bound antibodies due to the compensation of possible differences in biomolecule heights present at the micropatterned glass surface.

In a final step, we intended to work out optimum anti-bait antibody concentrations for live cell experiments (Figure 4C,D). Our standard working concentration, 10 µg/mL, was also used in previous studies under similar conditions and throughout the presented experiments. We could recently show that this concentration leads to >90% surface coverage within the pattern elements [43]. This might also explain the negligible increase in GFP-ErbB2 patterning when compared with higher antibody concentrations, such as 20 µg/mL (<c_10_> = 0.55 ± 0.10 vs. <c_20_> = 0.59 ± 0.12). Similar results were also obtained for tenfold lower antibody concentrations (1 µg/mL; <c_1_> = 0.49 ± 0.14), whereas a clear drop in GFP-ErbB2 enrichment in antibody-patterned areas was obtained for 0.1 µg/mL (<c_0.1_> = 0.19 ± 0.07). No specific bait patterning was detected below concentrations of 0.01 µg/mL antibody.

## 4. Conclusions

In this study, we describe a simple and straightforward method to produce highly condensed protein micropatterned glass substrates via µCP without the need of initial extensive chemical surface activation and modification. The presented approach has no need for clean room facilities and/or expensive equipment. PDMS stamps carrying a micron-scale array of features of interest can be easily fabricated from pre-manufactured wafers or can be directly purchased from various companies. Once the stamps are established, they can be reused a couple of times and, with the appropriate wafer size, large-area µCP can be realized. Nevertheless, the introduced method can be flexibly adapted to almost any substrate size. Most importantly, untreated substrates (e.g., glass or polymers such as COP, COC, etc.) are very cheap and, based on our experience, the AnteoBind^TM^ reagent can even be reused when recovered adequately after substrate incubation.

Altogether, this method represents a significant enhancement and simplification of existing µCP procedures and might further increase the accessibility of protein micropatterning for cellular biological research questions.

## Data Availability

The data presented in this study are available on request from the corresponding author.

## Supplementary Materials

The following supporting information can be downloaded at: https://www.mdpi.com/article/10.3390/bios12030140/s1: Figure S1: Simplified cross-section schemes of µCP procedure. Figure S2: µCP of different biomolecules.
