# Supplementary material for: A Simplified and Robust Activation Procedure of Glass Surfaces for Printing Proteins and Subcellular Micropatterning Experiments
Source: Biosensors (Basel). 2022 Feb 25;12(3):140. doi: 10.3390/bios12030140 (PMC8946821; doi:10.3390/bios12030140)
Supplement: Supplementary file 1 [file biosensors-12-00140-s001.zip › biosensors-1602820-supplementary.pdf]

biosensors

MDPI

Communication

A Simplified and Robust Activation Procedure of Glass Surfaces for Printing Proteins and Subcellular Micropatterning Experiments

Tina Karimian [1], Roland Hager [1], Andreas Karner [2], Julian Weghuber [1,3] and Peter Lanzerstorfer [1,*]

1 University of Applied Sciences Upper Austria, School of Engineering, 4600 Wels, Austria; tina.karimian@fh-wels.at; roland.hager@fh-wels.at; julian.weghuber@fh-wels.at; peter.lanzerstorfer@fh-wels.at
2 University of Applied Sciences Upper Austria, School of Engineering, 4020 Linz, Austria; andreas.karner@fh-linz.at
3 Austrian Competence Center for Feed and Food Quality, Safety & Innovation, FFoQSI GmbH, 3430 Tulln, Austria; julian.weghuber@ffoqsi.at
* Correspondence: peter.lanzerstorfer@fh-wels.at; Tel.: +43-50804-44402

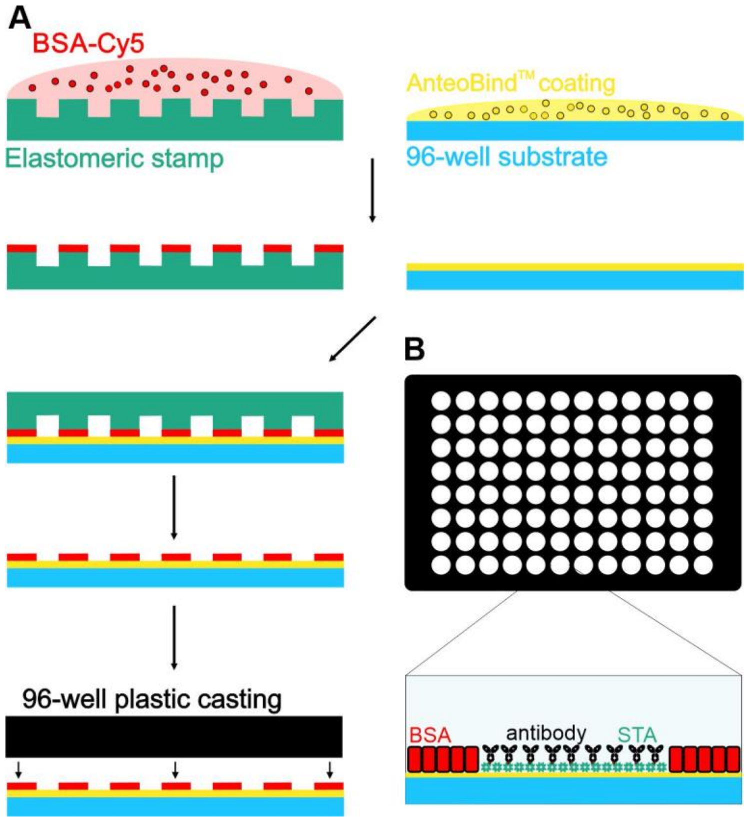

Figure S1. Cross-section sketches of µCP procedure. (**A**) A PDMS stamp is incubated with BSA (or BSA-Cy5) for surface passivation. At the same time, an uncoated clean glass substrate is covered with AnteoBind coating solution. Both, the stamp and the substrate, are washed and dried, followed by µCP (the stamp is laid onto the coated substrate by its own weight). After ripping of the stamp, the microstructured glass is finally bonded with a multi-well plastic casting. (**B**) Ready-to-use multi-well plate with micron-scale BSA grid for further live cell experiments. In a standard subcellular micropatterning experiment, single wells are further functionalized with streptavidin and biotinylated anti-bait antibodies prior cell seeding.

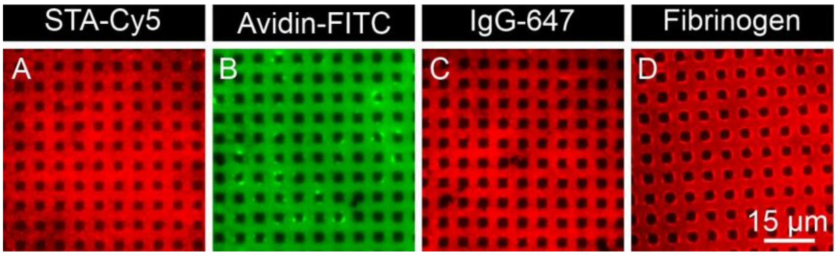

Figure S2. μCP of various biomolecules. Instead of printing BSA, different proteins were used to show the versatile binding properties of the surface coating. TIRF microscopy images show representative micron-scale protein grids of: (**A**) Cy5-conjugated streptavidin, (**B**) FITC-labeled avidin, (**C**) Alexa647-labeled anti-EGFR antibody and (**D**) biotin-labeled fibrinogen with subsequent incubation of STA-Cy5.
